# Highly Active Catalyst of Two-Dimensional CoS_2_/Graphene Nanocomposites for Hydrogen Evolution Reaction

**DOI:** 10.1186/s11671-015-1198-3

**Published:** 2015-12-21

**Authors:** Wei Xing, Yu Zhang, Qingzhong Xue, Zifeng Yan

**Affiliations:** School of Science, China University of Petroleum, Qingdao, 266580 People’s Republic of China; State Key Laboratory of Heavy Oil Processing, Key Laboratory of Catalysis, CNPC, China University of Petroleum, Qingdao, 266580 People’s Republic of China

**Keywords:** Hydrogen evolution reaction, Cobalt disulfide, Two-dimensional structure

## Abstract

Hydrogen evolution reaction (HER) by electrochemical water splitting using new promising non-precious metal catalysts shows great potential for clean energy technology. The design and fabrication of a high-performance electrode material based on cobalt disulfide/reduced graphene oxide (CoS_2_/RGO) nanocomposites is reported by a one-step hydrothermal method. Benefiting from its structural advantages, namely, large amount of exposed surface, fast charge transfer, and synergistic effect between CoS_2_ and RGO, the as-prepared nanocomposites are exploited as a catalyst for the HER. The results indicate that CoS_2_/RGO-5 % exhibits the best performance of hydrogen evolution and the smallest overpotential of 159 mV to achieve a 15 mA cm^−2^ current density, possessing the easiest releasing of hydrogen gas and the highest charge transfer rate, as well as remarkable stability.

## Background

Hydrogen, as a new type of renewable energy source, has attracted extensive concern due to its potential application in powering vehicles or electric devices. A promising method to produce hydrogen is electrocatalytic reduction of water via the hydrogen evolution reaction (HER) [1–3]. The platinum catalysts exhibit extraordinary activity in catalyzing the hydrogen evolution, but the high cost and scarcity seriously impede their practical applications. These limitations prompt the intensive investigations on inexpensive and earth-abundant electrocatalysts, e.g., metal sulfide [4], carbide [5], boride [6], and phosphide [7]. Many efforts have been taken to synthesize inorganic metal sulfur complexes to create analogs to these active materials. Particularly, MoS_2_ and WS_2_ have been researched as HER catalysts for their lower cost and higher stability than other metal derivatives [8–10]. On the other hand, carbon materials with excellent electrical conductivity, such as carbon nanotube and active carbons, were generally applied as supports of HER catalysts to enhance their stability and electrical conductivity [11]. Till now, only few reports were devoted to the study of 2D CoS_2_/graphene catalysts, which can expand the category of catalytic materials fitting for efficient HER [12].

Herein, we report a novel strategy to synthesize 2D cobalt disulfide/reduced graphene oxide (CoS_2_/RGO) nanocomposites with high HER activity by a hydrothermal method. In these nanocomposites, RGO serves as a matrix for the uniform growth of CoS_2_ nanoclusters. The presence of RGO facilitates both electrical conductivity and ionic transportation during the HER. Besides, the homogeneous dispersion of CoS_2_ nanoclusters on the surface of RGO could also incredibly increases the catalytic active sites [13]. Furthermore, the interaction between graphene and CoS_2_ can also inhibit the aggregation of CoS_2_ nanoclusters, resulting in improved cycling stability [14]. Benefiting from their high electrical conductivity, opened pore structure, excellent dispersion of CoS_2_, and positive synergistic effect between CoS_2_ and RGO, the CoS_2_/RGO nanocomposites exhibit excellent performance for HER process.

## Methods

### Materials

Natural graphite was provided by Qingdao Ruisheng Graphite Company. Cobalt(II) acetate tetrahydrate (Co(Ac)_2_·4H_2_O), thiourea (H_2_NCSNH_2_), ethyl alcohol, hydrogen peroxide (H_2_O_2_), potassium permanganate (KMnO_4_), concentrated sulphuric acid (H_2_SO_4_), and sodium nitrate (NaNO_3_) were purchased from Sinopharm Chemical Reagent Company and used without further purification. Nafion (5 wt%) water solution was purchased from Shanghai Yibang Technology Co., Ltd. Deionized water (DI) was prepared by a Millipore pure water system.

### GO Preparation and Purification

GO was synthesized by using modified Hummer’s method. In a typical synthesis, 1 g of graphite, 1 g of NaNO_3_, and 46 mL of H_2_SO_4_ were firstly mixed together in ice bath. Afterwards, 6 g of KMnO_4_ was added, and the suspension was heated to 35 °C. After vigorous stirring for 60 min, 80 mL of deionized water was added, and the temperature of solution was heated to 95 °C. Finally, 200 mL of water and 6 mL of H_2_O_2_ (30 wt%) was dropped into the solution, turning the color from dark brown to light yellow. Afterwards, the solution was filtered and washed thoroughly with deionized water. The filter cake was redispersed in water by strong mechanical agitation. After that, centrifugation was performed at 1500 rpm for four times until the solution is free of any visible particles. The supernatant was centrifuged at 8000 rpm for 20 min to remove tiny GO pieces. Finally, the precipitate was then redispersed in certain amount of water by sonication, resulting in an exfoliated GO solution (1 mg mL^−1^).

### Synthesis of CoS_2_/RGO Nanosheets

Co(Ac)_2_·4H_2_O (0.01 mol) and H_2_NCSNH_2_ (0.02 mol) were added to 60 mL of GO solution (containing 12.3 mg GO that is weighed by drying the GO solution). The mixture was then treated by sonication for about 30 min, giving a clear solution. Then, the solution was poured to a Teflon-lined autoclave (100 mL) and heated in an oven at 180 °C for 12 h. During this hydrothermal process, the GO was reduced to RGO. After being cooled to an ambient temperature, the resultant was separated by centrifugation, then washed with water and ethanol several times, and finally dried in an oven at 60 °C. For comparison, different GO dosages (36.9, 61.5, and 184.5 mg) were adopted in the preparation. The CoS_2_/RGO composites were denoted as CoS_2_/GO-*X* (*X* = 1, 3, 5, 15 %), where *X* is the mass ratio of GO and CoS_2_. Pure CoS_2_ was also synthesized under the same conditions in the absence of GO.

### Material Characterizations

The morphology of the CoS_2_/RGO nanosheets was observed by field emission scanning electron microscope (SEM, FEI Sirion 200) and transmission electron microscope (TEM, JEM 2010, JEOL, Japan). Crystal phase analysis was performed on a PANalytical X-ray diffractometer (XRD). Nitrogen sorption isotherms were determined at 77 K using a porosity analyzer (Tristar 3000, Micromeritics, USA). The Brunauer-Emmett-Teller (BET) area of the samples was obtained using the adsorption branch within the relative pressure (*P*/*P*_0_) ranging from 0.06 to 0.3. Pore size distribution (PSD) curve was obtained from the desorption branch by the BJH (Barrett-Joyner-Halenda) method.

### Electrochemical Measurements

All electrochemical studies were conducted on a CHI660D electrochemical work station in a three-electrode setup using the as-prepared samples as working electrode, a calomel electrode as the reference electrode, and a platinum foil as a counter electrode in 0.5 M H_2_SO_4_ electrolyte. The oxygen dissolved in the electrolyte was removed by purging with nitrogen for 10 min prior to the experiments. Linear sweep voltammetry was used to determine the electrocatalytic activities of the as-prepared samples (with an active area of 0.5 cm^2^) towards the HER at room temperature. HER measurement was also performed using a carbon counter electrode to preclude any Pt contamination. The results showed that the polarization curves from either counter electrode are almost the same. All the potentials were referred to the reversible hydrogen electrode (RHE) without specification.

## Results and Discussion

**Fig. 1:**
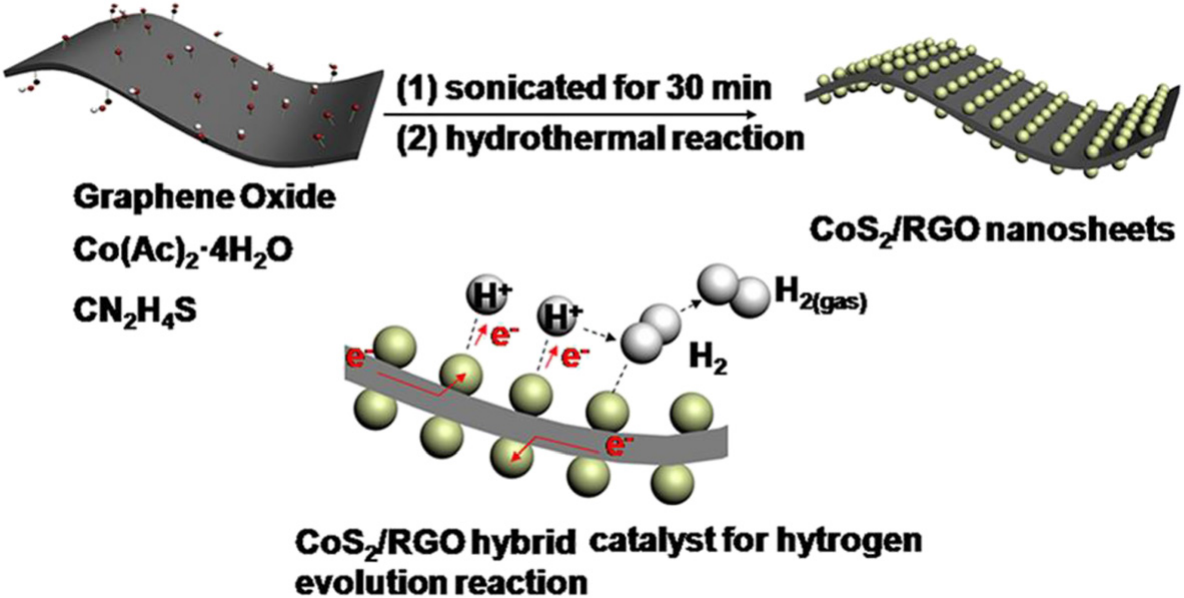
Synthesis strategy and hydrogen evolution process of the CoS_2_/RGO nanocomposites

The strategy for the synthesis of the CoS_2_/RGO nanosheets is illustrated in Fig. 1. CoS_2_ nanoclusters were in situ grown on the RGO surface through a facile hydrothermal process. In the structure of CoS_2_/RGO nanocomposites, the highly conductive graphene and the 2D structure of the composites are beneficial for electron transfer and proton transport, respectively. This is supposed to facilitate the proton reduction at the catalyst/electrolyte interface. Besides, the high dispersion of CoS_2_ nanoclusters on the surface of graphene will increase drastically the catalytic active sites for the hydrogen evolution reactions [15–17].

**Fig. 2:**
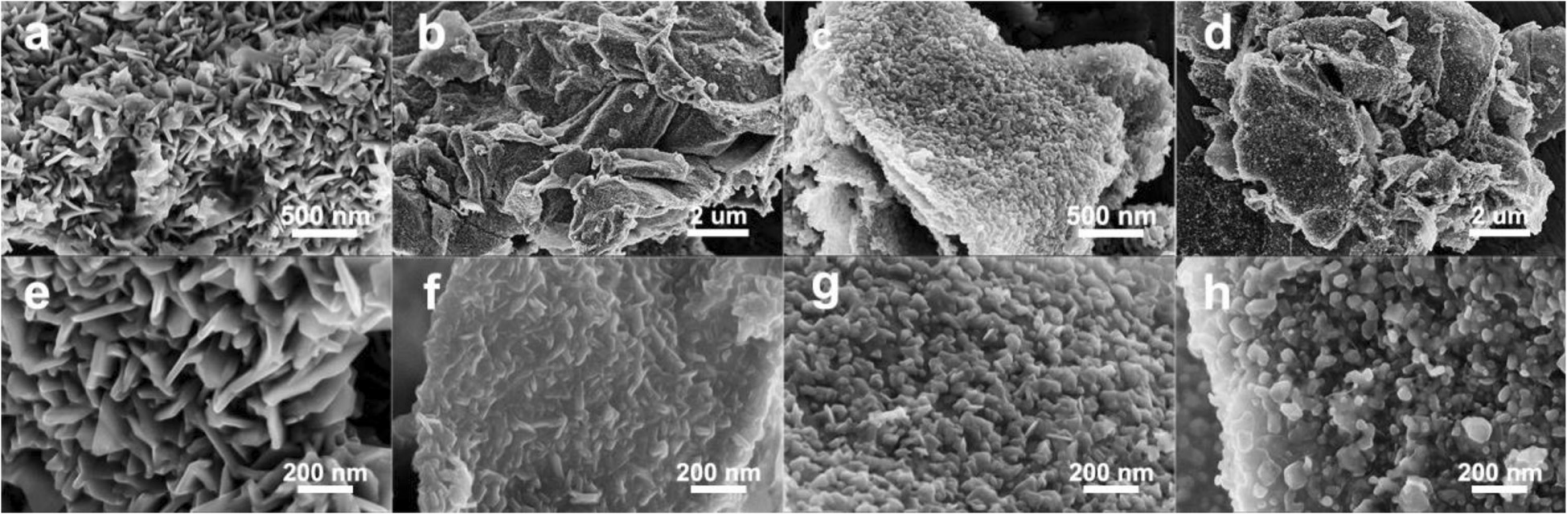
SEM images of CoS_2_/RGO-1 % (**a**, **e**), CoS_2_/RGO-3 % (**b**, **f**), CoS_2_/RGO-5 % (**c**, **g**), and CoS_2_/RGO-15 % (**d**, **h**)

**Fig. 3:**
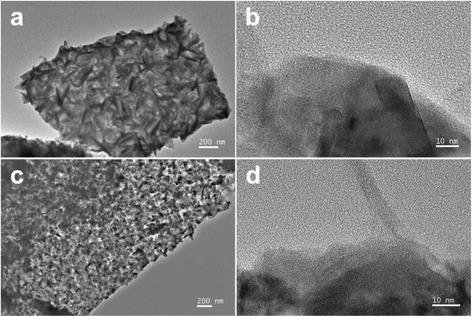
TEM images of CoS_2_/RGO-1 % (**a**, **b**) and CoS_2_/RGO-5 % (**c**, **d**)

As shown in Fig. 2a–d, CoS_2_ nanoclusters grow uniformly on both sides of the graphene sheets with a size of several micrometers, and the size of nanoclusters is about 50–80 nm. It also can be found from Fig. 2e–h that the morphology of CoS_2_ nanoclusters changes from vertically dispersed nanosheets to uniformly distributed nanoparticles with the increase of GO dosage. This is because the amount of CoS_2_ is only enough to form crystal nucleuses at the large dosage of GO. Higher GO dosage would be beneficial for better dispersion of CoS_2_ nanoclusters, contributing to more exposed CoS_2_ surface for HER. The high-resolution TEM images demonstrate that most of the CoS_2_ nanosheets stand vertically on graphene for CoS_2_/RGO-1 % sample (Fig. 3a, b). When the GO dosage was increased to 5 wt%, the shape of CoS_2_ turns from nanosheets to nanoparticles. (Fig. 3c, d). The SEM and TEM observations agree with each other.

**Fig. 4:**
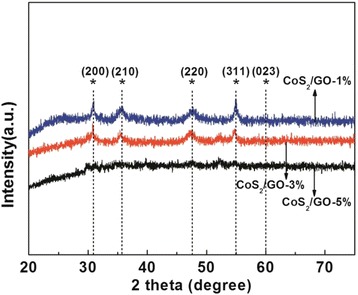
XRD patterns of CoS_2_/RGO-1 %, CoS_2_/RGO-3 %, and CoS_2_/RGO-5 %

The crystal phase of the CoS_2_/RGO composites was detected by X-ray diffraction, as is presented in Fig. 4. It is shown that all the peaks are attributed to the cubic CoS_2_ phase (JCPDS number 41-1471), which should be an active phase for HER. The peak intensity decreases with the rise of GO dosage because of better dispersion of CoS_2_ on more RGO. No diffraction peaks for RGO can be hardly detected in the spectrum due to its low content.

**Fig. 5:**
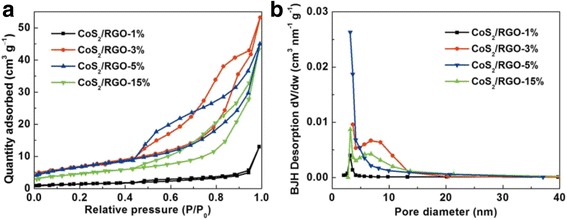
Nitrogen adsorption-desorption isotherms (**a**) and PSD curves (**b**) of CoS_2_/RGO-1 %, CoS_2_/RGO-3 %, CoS_2_/RGO-5 %, and CoS_2_/RGO-15 %

The pore texture of the as-prepared samples was determined by nitrogen sorption measurement at 77 K. As shown in Fig. 5a, all the CoS_2_/RGO nanocomposites show typical type IV isotherm and obvious hysteresis loop, revealing their mesoporous nature. The BET specific surface area of CoS_2_/RGO-5 % is 26.2 m^2^g^−1^ (Table 1), which is the highest among these samples. The larger porosity of CoS_2_/RGO-5 % sample is also manifested by the PSD curves in Fig. 5b. The large surface area and pore volume are also supposed to facilitate its catalytic activity to HER.

**Table 1:** Surface area and pore volume of the as-prepared catalysts

Samples	*S*_BET_^a^	*S*_Micro_^b^	*S*_Meso_^c^	Pore volume^d^
	m^2^ g^−1^	m^2^ g^−1^	m^2^ g^−1^	cm^3^ g^−1^
CoS_2_/RGO-1 %	5.0	0.7	4.3	0.021
CoS_2_/RGO-3 %	24.9	0.9	24	0.082
CoS_2_/RGO-5 %	26.2	0.6	25.6	0.087
CoS_2_/RGO-15 %	16.4	1.1	15.3	0.067

^a^BET specific surface areas

^b^Micropore surface areas calculated by t-plot method

^c^Mesopore surface areas equal to *S*_BET_ minus *S*_Micro_

^d^Total pore volume calculated at the relative pressure of 0.99

**Fig. 6:**
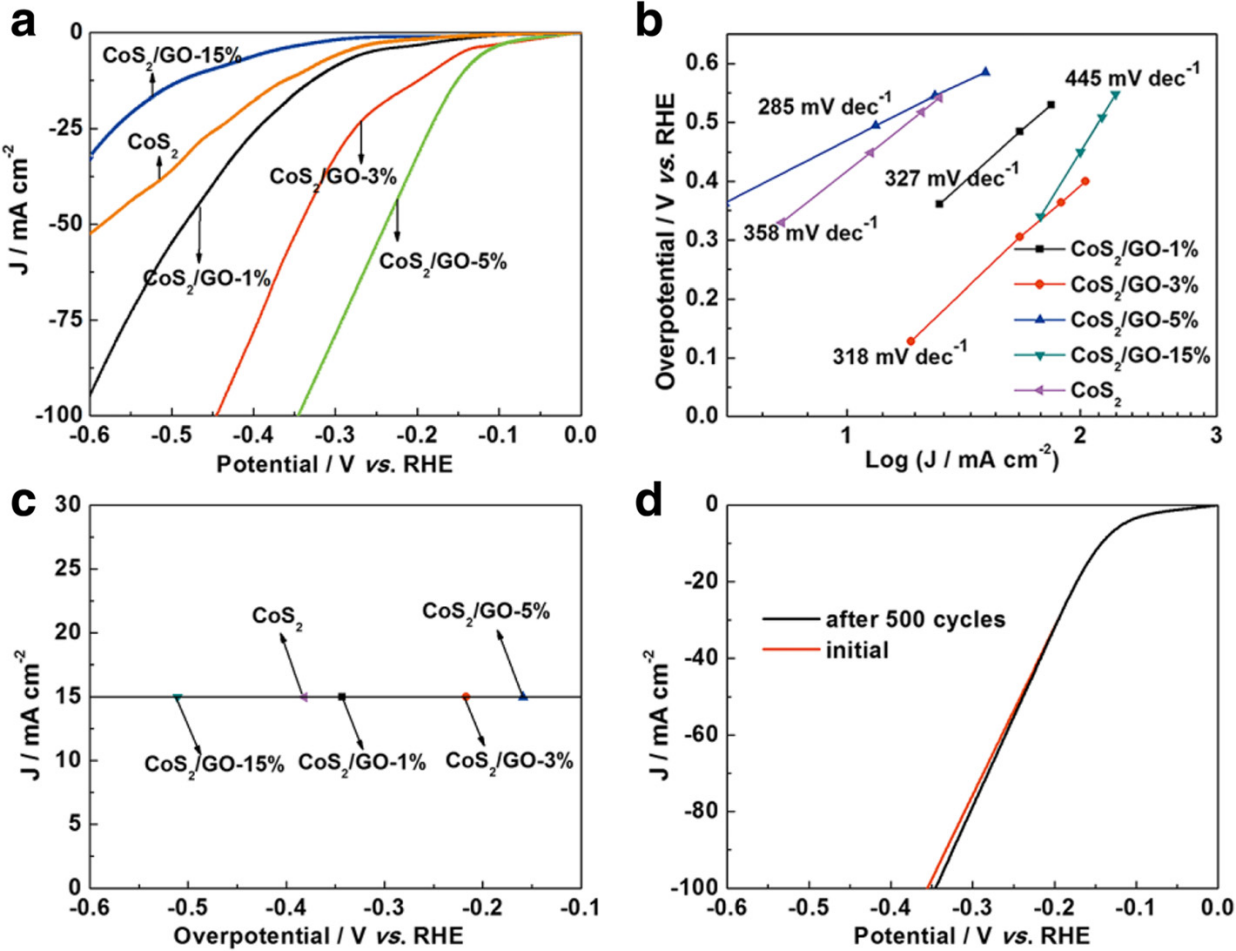
**a** The HER polarization curves, **b** corresponding Tafel plots, and **c** the HER current density at 15 mA cm^−2^ versus overpotential. **d** Stability test for the CoS_2_/RGO-5 % electrode

The as-prepared CoS_2_/RGO catalyst coated on glassy carbon was applied as a working electrode for the HER. Pure CoS_2_ was also evaluated for comparison. As shown in Fig. 6a, the polarization curves is measured in 0.5 M H_2_SO_4_ electrolyte with a voltage sweep rate of 5 mV s^−1^. The polarization curve of CoS_2_/RGO-5 % sample shows a small overpotential of 143, 173, and 346 mV at the current densities of 10, 20, and 100 mA cm^−2^, respectively, indicating a good HER activity. In contrast, pure CoS_2_ exhibits overpotential of 414 mV at a current density of 20 mA cm^−2^, which is apparently larger than the overpotential detected for CoS_2_/RGO composites, suggesting its worse HER activity. On the other hand, at the potential of 300 mV, the CoS_2_/RGO-5 % shows a large cathodic current density of 78.8 mA cm^−2^ which is about nine times that of the CoS_2_/RGO-1 % electrode (9.0 mA cm^−2^) and far larger than the pure CoS_2_ electrode (6.0 mA cm^−2^). The Tafel slope of CoS_2_/RGO-5 % in Fig. 6b is 285 mV dec^−1^, thus leading to a faster increment of the HER rate with increasing overpotential. The polarization curve recorded on CoS_2_/RGO-5 % displays the lowest overpotential of 159 mV to get a 15 mA cm^−2^ current density (Fig. 6c), which is smaller than those of the other samples. Therefore, the CoS_2_/RGO-5 % with the smallest slope shows the highest activity in the HER. The excellent catalytic performance of CoS_2_/RGO-5 % is due to its large surface area, which leads to high dispersion of CoS_2_ nanoparticles and more active sites. Compared with pure CoS_2_, the better performance of CoS_2_/RGO composites (1, 3, 5 %) also indicates the positive synergistic effect between CoS_2_ and RGO for HER.

To evaluate the stability of the CoS_2_/RGO-5 % catalyst, the electrode is operated continuously for 500 cycles of potential scans (scan rate 5 mV s^−1^). The results show that there is no apparent recession of the activity, demonstrating robust catalytic durability of the CoS_2_/RGO-5 % catalyst in acidic electrolyte (Fig. 6d). This should be attributed to the interaction between CoS_2_ and RGO, which stabilize the CoS_2_ nanoparticles in HER process.

## Conclusions

In conclusion, we have proposed a novel method to synthesize active CoS_2_/RGO nanocomposites by hydrothermal treatment. The obtained sample can be employed as a catalytic material for the hydrogen evolution reactions. The synergistic effect of CoS_2_ and RGO contributes to the good HER activity of these nanocomposites. Besides, this effective synthesis method could be extended to manufacture more promising electrode materials for fuel cell, Li-ion or Na-ion batteries, and supercapacitors.
